# Supplementary material for: Fast Mass-Production of Medical Safety Shields under COVID-19 Quarantine: Optimizing the Use of University Fabrication Facilities and Volunteer Labor
Source: Int J Environ Res Public Health. 2020 May 14;17(10):3418. doi: 10.3390/ijerph17103418 (PMC7277794; doi:10.3390/ijerph17103418)
Supplement: Supplementary file 1 [file ijerph-17-03418-s001.pdf]

Supplementary Materials:

S1. Examples of medical face shields available at the markets of USA and UK. Industrial products.

N	Appearance	Producer	Price per 1 pcs	Material/Weight, g
1	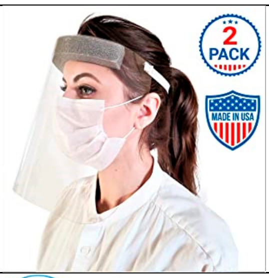	Dexter, Minnesota, USA	15 USD	Undisclosed (Elastic Band and Comfort Sponge)/368.5
2	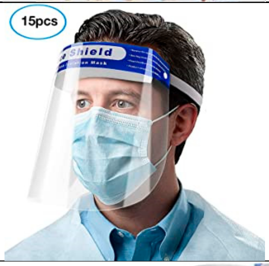	China	1.26 USD	High-quality optical and distortion-free 7mm polyester film/ Undisclosed
3	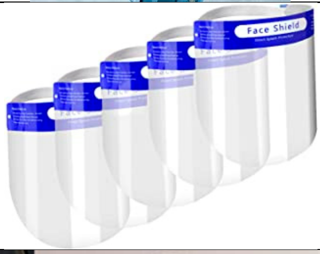	China	2.60 USD	PET visor + PC frame/80 …141 g
4	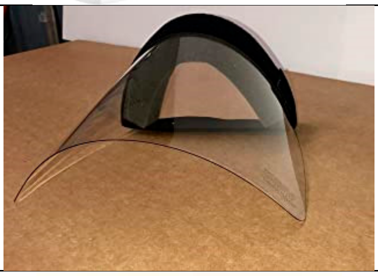	Plastifab Inc. AZ, USA	26.50 USD	Polycarbonate + PETG/173 g
5	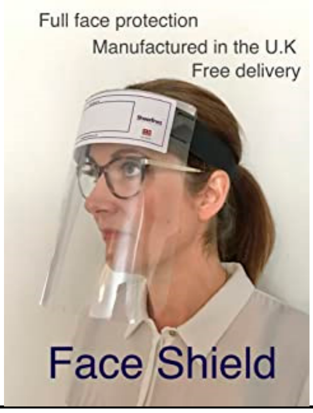	UK	7,50 GBP	PPE/Undisclosed

S2. Technical Data Sheet and Assembling Instructions.

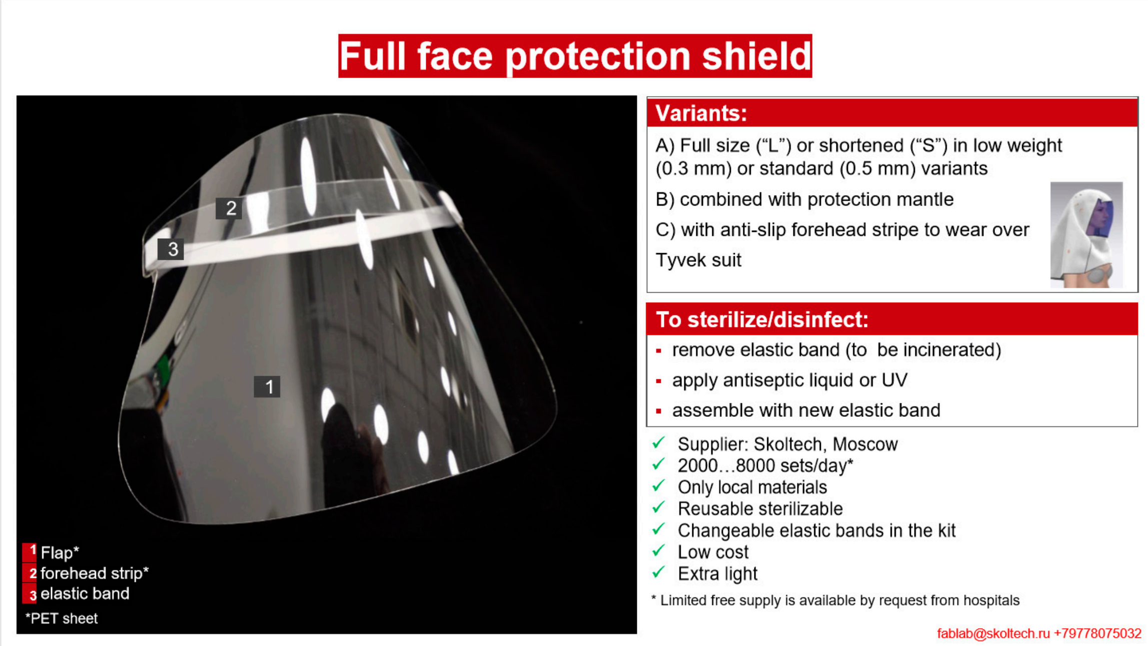

(a) Technical Data Sheet.

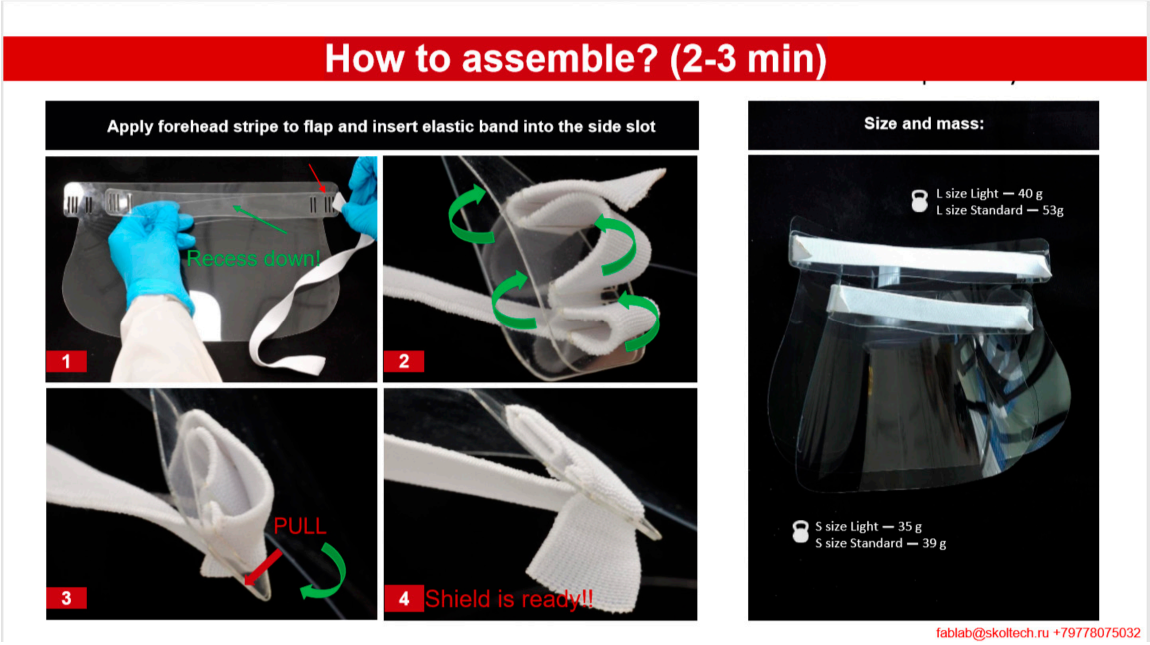

(b) Assembling Instructions

S3. Institutions that performed tests of Face Medical Shields

1. MEDSI group clinics. Moscow City, https://medsi.ru/services/statsionar/
2. Federal State Autonomous Educational Institution of Higher Education I. M. Sechenov First Moscow State Medical University of the Ministry of Health of the Russian Federation (Sechenov University) https://www.sechenov.ru/univers/about_lecturer/56202/
